# A New Ochratoxin A Biodegradation Strategy Using *Cupriavidus basilensis* Őr16 Strain

**DOI:** 10.1371/journal.pone.0109817

**Published:** 2014-10-10

**Authors:** Szilamér Ferenczi, Mátyás Cserháti, Csilla Krifaton, Sándor Szoboszlay, József Kukolya, Zsuzsanna Szőke, Balázs Kőszegi, Mihály Albert, Teréz Barna, Miklós Mézes, Krisztina J. Kovács, Balázs Kriszt

**Affiliations:** 1 Institute of Experimental Medicine, Laboratory of Molecular Neuroendocrinology, Budapest, Hungary; 2 Szent István University, Department of Environmental Protection and Safety, Gödöllő, Hungary; 3 Central Environmental and Food Science Research Institute, Department of Microbiology, Budapest, Hungary; 4 Soft Flow Hungary R&D Ltd., Pécs, Hungary; 5 CEVA Phylaxia Ltd, Budapest, Hungary; 6 University of Debrecen, Department of Genetics and Applied Microbiology, Debrecen, Hungary; 7 Szent István University, Department of Nutrition, Gödöllő, Hungary; Northwestern University Feinberg School of Medicine, United States of America

## Abstract

Ochratoxin-A (OTA) is a mycotoxin with possibly carcinogenic and nephrotoxic effects in humans and animals. OTA is often found as a contaminant in agricultural commodities. The aim of the present work was to evaluate OTA-degrading and detoxifying potential of *Cupriavidus basilensis* ŐR16 strain. *In vivo* administration of OTA in CD1 male mice (1 or 10 mg/kg body weight for 72 hours or 0.5 mg/kg body weight for 21 days) resulted in significant elevation of OTA levels in the blood, histopathological alterations- and transcriptional changes in OTA-dependent genes (*annexinA2*, *clusterin*, *sulphotransferase* and *gadd45* and *gadd153*) in the renal cortex. These OTA-induced changes were not seen in animals that have been treated with culture supernatants in which OTA was incubated with *Cupriavidus basilensis* ŐR16 strain for 5 days. HPLC and ELISA methods identified ochratoxin α as the major metabolite of OTA in *Cupriavidus basilensis* ŐR16 cultures, which is not toxic *in vivo*. This study has demonstrated that *Cupriavidus basilensis* ŐR16 efficiently degrade OTA without producing toxic adventitious metabolites.

## Introduction

Ochratoxin-A (OTA) is a hazardous mycotoxin produced by number of *Aspergillus* and *Penicillium* species [1]. Mycotoxins are extracellular secreted secondary metabolites of moulds that are harmful or toxic to animals and humans [2]. The chemical structure of OTA molecule (*N*-{[(3*R*)-5-chloro-8-hydroxy-3-methyl-1-oxo-3,4-dihydro-1*H*-isochromen-7-yl]carbonyl}-L-phenylalanine) includes a β-phenylalanine-dihydroisocoumarine derivative, which is very stable at high temperature and resistant to hydrolysis, hence processing of raw materials in feed and food industry does not eliminate the OTA and the toxin remains intact in the end-products. The OTA is often found as a contaminant in cereal grains or other crops and plant products such as red wine, coffee beans, peanuts, cocoa beans, and different spices [3]-[5]. Dried Distillers Grains with Solubles (DDGS) remaining after bioethanol production from maize is a valuable protein source for animal nutrition, but high mycotoxin content of DDGS limits its application [6]. On the other hand, the mycotoxin is also capable to accumulate in several animal-derived food products (meat, egg, blood and milk products) [7].

Chronic OTA exposure is the major causative chemical of mycotoxin-induced porcine nephropathy [8], [9] and Balkan endemic nephropathy (BEN) in humans [10]-[13]. The OTA-induced nephropathy is characterized by degeneration of epithelial cells in the renal proximal tubules, glomerulus degeneration in renal cortex area and interstitial fibrosis resulting in polyuria and various changes in hematological and biochemical parameters [14]. On the other hand, chemical structure of OTA shows similarity to the amino acid phenylalanine, thus the toxin is able to interrupt the protein synthesis [15], [16]. Influence of OTA as a causal chemical substance of different types of cancer such as renal adenocarcinoma and liver tumor have been described in laboratory rodents and in humans, as well [17], [18]. OTA affects the expression of several genes related to cell damage, apoptosis, cellular stress, such as Growth arrest and DNA-damage-inducible proteins (*gadd45* and *gadd153*), *annexins*, *sulfotransferase* and *clusterin* [19].

Several strategies can be used to reduce OTA levels in animal feed and human food. The most important are preventive methods since they avoid the contamination of commodities in the first place. However, fully implemented Hazard Analysis and Critical Control Points (HACCP) schemes are rare, and when the individual measures fail or are not in place, OTA remains in feed and food products. Decontamination or detoxification procedures can be used to remove or to reduce OTA levels. Remediation processes are often used to eliminate, reduce or avoid the toxic effects of OTA. The widely used physical adsorbents have some disadvantages including limited efficiency, high cost and nonspecific binding of some important nutrients, such as vitamins or minerals and therapeutic agents. Biological methods have been considered as an alternative to physical and chemical treatments. The biodegradation is the most promising approach to control mycotoxins and these methods are important postharvest strategies to protect the animal and consumer health.

Several enzymes may be involved in the microbiological degradation of OTA. However, little information is available and very few have been purified and characterized. The first reported protease able to hydrolyze OTA was carboxypeptidase A (CPA) (EC 3.4.17.1) from bovine pancreas [20].

Previously more than fifteen species of bacteria [21] have been shown to degrade OTA – but not yet any *Cupriavidus* species – and many species of fungi have also been reported. However, vast majority of this biodegradation by microorganisms cannot be regarded as detoxification since the toxicity after the biotransformation was not elucidated. To develop a bacterial strain to efficient biodegradation of OTA, selection of the most active and environmentally safe microbes is required. The aim of the present work was to evaluate the OTA-degrading and detoxifying potential of a *Cupriavidus basilensis* strain, which mycotoxin- degrading ability was presumable based on its genome project [22].

Traditional chemical analysis and immunochemical methods are unable to detect the toxic effect of all potential degradation products; therefore it is important to monitor toxicity *in vivo*. Moreover, the European Food Safety Authority reported that contamination of animal feed by mycotoxins may be reduced by mycotoxin-detoxifying agents but the additive effects of the resulting metabolite(s)/degradation products(s) must also be monitored in appropriate toxicological studies. Thus, development of *in vivo* studies is essential to investigate biodegradation and detoxification efficiency directly on the renal cortical area, the most sensitive organ for OTA in animals and humans, as well. For this purpose the application of a rodent based *in vivo* toxicological experiment can be the most suitable approach, in which two markers are analysed: alterations in the kidney and spleen weight and changes in the expression of OTA-affected genes in the kidney tissue [19]. This method in combination with analytical and immunochemical techniques was applied to analyse OTA and residuals toxicity after biodegradation.

## Materials and Methods

### 2.1 Reagents

Ochratoxin-A (OTA) (Fermentek, Israel), methyl- methanesulfonate (MMS) (Sigma-Aldrich Co., USA), OTalpha standard (Biopure, cat. number: BRM S02053, lot. number: L12503A), dimethyl-sulfoxid - DMSO (Sigma-Aldrich Co., USA), Tris-(hydroximethyl)- aminomethane, Tris (Sigma_Aldrich, USA), modified Luria-Bertani (LB) medium (1 g triptone, 0,5 g yeast extract, 0,9 g NaCl in 1000 ml distilled water) were used.

### 2.2 Bacterial strain and culture conditions in the biodegradation test

The strain *Cupriavidus basilensis* ŐR16, was isolated from a Hungarian pristine soil sample. It was identified by molecular taxonomy as *C. basilensis* and deposited in the National Collection of Agricultural and Industrial Microorganisms (NCAIM BO2487). Cells were streaked on LB agar plates and incubated at 28°C for 72 h. Single colonies were inoculated into 50 ml liquid LB medium and incubated at 170 rpm at 28°C for 72 h. The optical density of the cultures was adjusted to 0.6 (OD_600_ = 0.6) and 50 ml was added to 1350 ml sterile modified LB medium to which OTA (28 mg OTA was dissolved in the 1400 ml modified LB medium) had been added reaching a 20 mg/l final concentration. A no inoculated control with 20 mg/l OTA content and a control culture without OTA was applied. Control culture was essential since in toxicity testing animals are presumably sensitive to metabolic by-products of the *C. basilensis* ŐR16 strain. Samples were incubated (170 rpm, 28°C) for 5 days. One-millilitre samples from the flasks were removed at the 1^st^, 2^nd^, 3^rd^, 5^th^ day, centrifuged at 25,000 × g at 4°C for 20 min and both supernatant and pellet were stored at −20°C until further use. At the 5^th^ day (end-point) of the experiment the entire pellet material was removed (25,000 × g at 4°C for 20 min) and the supernatant was concentrated by a factor of 100 on an Edwards Micromodulyo lyophilisator and the lyophilised supernatant was used in the animal experiments. Remaining OTA concentrations in the supernatant were analyzed by High Performance Liquid Chromatography (Wessling Hungary Ltd., Hungary) and the supernatant and pellet were analysed by ELISA (Soft Flow Ltd., Hungary).

### 2.3 Analysis of samples for remaining toxin concentration

#### 2.3.1 Enzyme-linked immunosorbent assay (ELISA)

The OTA toxin concentrations at the zero point in the supernatant and also in the pellet on the 1^st^, 2^nd^, 3^rd^ and 5^th^ day of the degradation experiment were determined by TOXI-WATCH ELISA kit (Cat#301051, Soft Flow Hungary R& D Ltd., Hungary) according to the manufacturer's specifications. Assay range: (0.1375 ng/ml–44 ng/ml, limit of detection: 0.130 ng/ml). 0.1M PBS buffer with 3% sodium-hydrogen-carbonate NaHCO_3_, (4∶1 v/v%) was used for sample dilution, while standards contain the same solution (PBS/3% NaHCO_3_ 4∶1 ratio).

The 201052-5G9 (Soft Flow Hungary Ltd., Hungary) monoclonal antibody specifically binds to the mycotoxin OTA. The immunogen used to generate the hybridoma 5G9 was OTA-BSA conjugate. The 201052-5G9 antibody cross-reacts with ochratoxin-B (9.3%). Measurements were carried out in triplicate and the measurements were performed on Thermo Scientific Multiskan EX photometric microplate absorbance reader.

#### 2.3.2 High-performance liquid chromatography (HPLC)

HPLC analyses were carried out by Wessling Hungary Ltd., an accredited laboratory, using a HPLC series 1100 from Agilent Technologies, USA.

The supernatant collected at the beginning and on the 1^st^, 2^nd^, 3^rd^, 5^th^ day of the degradation experiment, was analyzed for OTA and its derivative OT-α. The bacterial pellet was suspended in 1 ml methanol and centrifuged (3,000 × g for 10 min at <10°C), then the supernatant was analysed for OTA. Results for degradation potential were corrected with the pellet analysis.

Protocols for the immuno-affinity column cleaning, derivatization, LC separation and fluorescence detection of the compounds were carried out according to European Standard (EN) and International Organization for Standardization (ISO) (EN ISO 15141-1∶2000 standard) for OTA. For the determination of OTα, the column, eluent composition and detection parameters were modified (see in Supplementary Materials, Table S1). HPLC measurements were carried out in triplicates.

### 2.4 Animals

Adult, 7–9 week old, male CD1 mice (from the colony breed at the Institute of Experimental Medicine, Budapest) were used. Animals had free access to rodent food and water and were maintained under controlled conditions (temperature, 21±1°C; humidity, 65%; light-dark cycle, 12-h light/12-h dark, lights on at 07∶00). All procedures were conducted in accordance with the guidelines set by the European Communities Council Directive (86/609 EEC) and the protocol was approved by the Institutional Animal Care and Use Committee of the Institute of Experimental Medicine, Budapest Hungary (permit number: PEI/001/35-4/2013).

### 2.5 Measuring of the blood OTA concentrations by ELISA

Extraction of OTA was carried out from 50 µl plasma sample with 100 µl chloroform (Sigma-Aldrich Co., USA) and 10µl 0.1 M H_3_PO_4_. The mixture was vortexed and incubated on Bio RS-24 vertical rotator (Biosan) rotating 25 rpm for 20 min then vortexed again and centrifuged for 10 min at 10000 × g. The lower phase was transferred to a microcentrifuge tube, vortexed with 100 µl 3% (w/v) NaHCO_3_ solution, and incubated on the vertical rotator (25 rpm, 10 min). The phases were let to split and the upper phase was used for further measurements.

The OTA concentration of the extract was measured by Toxi-Watch OTA ELISA Kit (Cat#301051, Soft Flow Hungary Ltd., Hungary). Measurements were performed on a Thermo Scientific Multiskan EX photometric microplate absorbance reader. Measurements were carried out in triplicate. The recovery was 77.6 ±5.42 % as was measured with 20 ng/ml OTA spiked blood plasma samples.

### 2.6 Animal treatment

Male CD1 mice were housed and treated according to OECD guideline for the testing of chemicals No. 407 with full access to food and drinking water. Body weight, food and water consumption was recorded daily. OTA was dissolved in DMSO and a stock solution in 100 mg/ml OTA concentration was prepared, stored at 4°C. Thereafter this master solution was diluted into sterile drinking water containing 10 mM Tris (pH 8) to reach the required experimental OTA dose. OTA and vehicle solutions were administered daily via oral gavage (200µl/animal) in the morning hours.

For testing OTA, three different dosage groups (n = 7-10/group) were formed. For acute tests 1 mg/kg bw and 10 mg/kg bw sacrificed after 72 h; for chronic test 0.5 mg/kg bw, sacrificed after 21 day. Sterile drinking water supplemented with 10 mM Tris and equal concentration of DMSO with high dose OTA group in acute experiment and equal concentration of DMSO with chronic experiment were applied as control. As genotoxic control, MMS was used in 100 mg/kg bw dose for the 72 h experiment and 40 mg/kg bw dose for the 21 day experiment [23].

For testing OTA detoxification by *C. basilensis* Őr16, samples and controls originated from the degradation experiment were applied (section 2.2). The lyophilized supernatant from the biodegradation experiment with *C. basilensis* Őr16 strain was dissolved in 11.2 ml sterile tap water containing Tris and DMSO following the same procedures that used in high OTA content preparation (10 mg/kg bw). The theoretical OTA concentration of this solution is 2.5 mg/ml, calculated by initial OTA quantity (28 mg OTA). This stock solution was used for the treatment of high OTA deg group (group treated with theoretical 10 mg/kg body weight Ochratoxin A). Forty µl from this solution was added to each 10g body weight (0.1 mg theoretical OTA quantity). Following dilutions were made from this stock solution for the lower OTA deg groups treatment (group treated with 1 mg/kg body weight and 0.5 mg/kg bw Ochratoxin A) by using sterile tap water containing 10 mM Tris (pH 8) and balanced DMSO. Doses were calculated from the animal weights and administered via oral gavages.

The animal treatment with intact Ochratoxin A and the biodegradation experiment were carried out from same OTA batch. Animals were decapitated, trunk blood collected on ice, spleen and kidney were removed and their weights were measured. Kidney was cut longitudinally resulting in half kidneys. One half was fixed in 10% buffered formalin solution for histological examination. From the other half of the kidney, the cortex and medulla were separated and frozen immediately in dry ice and stored at −80°C to prevent RNA degradation.

### 2.7 Quantitative Real-Time PCR

Frozen kidney cortex tissue samples were homogenized by IKA Ultra Turrax in TRI Reagent Solution (Ambion, USA) and total RNA was isolated with QIAGEN RNeasy Mini Kit (Qiagen, USA) according the manufacturer's instruction. To eliminate genomic DNA contamination DNase I treatment were used and 100 µl Rnase-free DNase I (1 unit DNase) (Thermo Scientific, USA) solution was added. Sample quality control and the quantitative analysis were carried out by NanoDrop (Thermo Scientific, USA). Amplification was not detected in the RT-minus controls. The cDNA synthesis was performed with the High Capacity cDNA Reverse Transcription Kit (Applied Biosystems, USA). Primers for the comparative Ct experiments were designed by Primer Express 3.0 Program. The primers (Microsynth, Balgach) were used in the Real-Time PCR reaction with Fast EvaGreen qPCR Master Mix (Biotium, USA) on ABI StepOnePlus instrument was listed in Table S2.

List of the genes: peptidylprolyl isomerase A (*ppia*, NM_008907), annexin A2 (*anxa2*, NM_007585), clusterin (*clu*, NM_013492), DNA-damage inducible transcript 3 (*gadd153*, NM_007837), growth arrest and DNA-damage-inducible 45 alpha (*gadd45a*, NM_007836) and sulfotransferase (*sult1c2*, NM_026935).

The gene expression was analyzed by ABI Step One 2.1 program. The amplicon was tested by Melt Curve Analysis on ABI Step OnePlus Instrument. Experiments were normalized to *ppia* (peptidylprolyl isomerase A) expression [24].

### 2. 8 Histology

Formalin fixed kidney samples were processed and embedded in paraffin using the standard protocol. Sections of 4 µm were stained with haematoxylin and eosin. Slides were analysed by an expert veterinary pathologist in a blinded manner.

### 2.9 Statistical analysis

Data are expressed as means ± SD. The data were first subjected to a Kolmogorov-Smirnov normality test. Data passing this test, were analyzed by One way ANOVA followed by the Tukey's *post hoc* test. Data showed non-Gaussian distribution, the Kruskal-Wallis test was used. Statistical analysis was performed using GraphPad PRISM version 6 software (GraphPad Software, USA). *P* ≤ 0.05 was considered significant.

## Results

### 3.1 Ochratoxin-A biodegradation by *Cupriavidus basilensis* ŐR16

**Figure 1:**
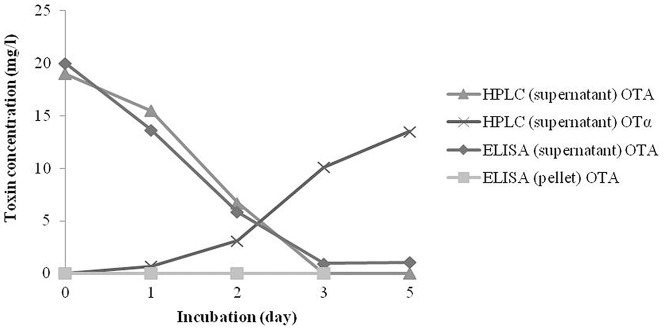
Ochratoxin-A biodegradation by *Cupriavidus basilensis* ŐR16 during 5 day incubation. Continuous decrease of the OTA concentration is detected in the supernatant and pellet, while OTα concentration is increasing. Abbreviations: HPLC (supernatant OTA) – OTA concentration in the supernatant originated from the degradation experiment measured by HPLC, HPLC (supernatant OTα) – OTα concentration in the supernatant originated from the degradation experiment measured by HPLC, ELISA (supernatant) OTA – OTA concentration in the supernatant originated from the degradation experiment measured by ELISA, ELISA (pellet) OTA – OTA concentration in the pellet originated from the degradation experiment measured by ELISA. Measurements were carried out in triplicate, SD>3%.

The biodegradation ability of *Cupriavidus basilensis* ŐR16 strain to detoxify OTA was monitored by different analytical approaches. The HPLC and ELISA results are summarised in Fig. 1, where samples originating from the OTA degradation experiments (1^st^, 2^nd^, 3^rd^, 5^th^ day) are indicated as a function of time. OTA concentration in the no inoculated control remained 20 mg/l during the incubation (5 days) measured by HPLC and ELISA. In the bacterial pellet ELISA detected lower than 0.004% residual OTA of the original concentration (20 mg/l). The first day of incubation *Cupriavidus basilensis* ŐR16 cells degraded 0.15% of the initial OTA content, but this OTA content reduced by the 5^th^ day below 0.004%, thus OTA elimination from the matrix was attributed to metabolic activity. OTA content in the supernatants reduced continuously during the 5-day incubation and the OTA was completely degraded (94% decrease measured by ELISA, 100% decrease by HPLC). Based on the HPLC results OTA was metabolized to OTα, since OTα content increased in parallel by OTA decrease.

### 3.2 Water, food consumption and body weight change of mice

Food and water consumption did not change significantly during acute or chronic OTA administration with the supernatant samples from biodegradation study. Body weight of the treated animals in either experiment also did not alter significantly.

### 3.3 Blood OTA content in acute and chronic toxicities

**Figure 2:**
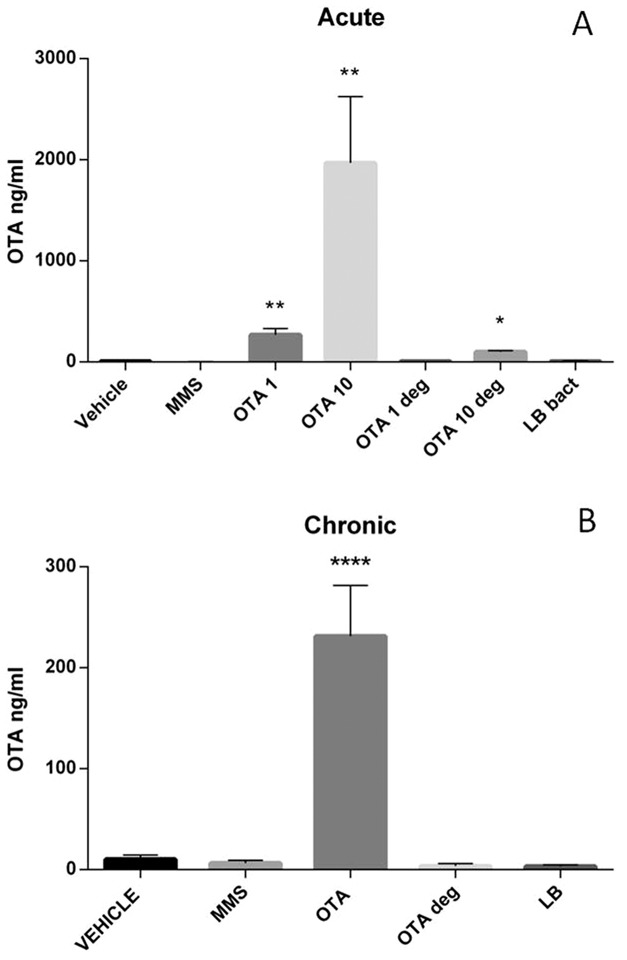
OTA concentration in the plasma (ng/ml) analyzed by ELISA after 72 hours of treatment (**A**). OTA 1 and OTA 10 groups show elevated OTA concentrations in blood plasma. The OTA 10 deg group shows increased OTA content. Abbreviations: MMS- Group treated with methyl methanesulfonate, OTA 1 and OTA 10- Groups treated with 1 and 10 mg/kg body weight Ochratoxin A, OTA 1 deg and OTA 10 deg- Groups treated with 1 and 10 mg/kg body weight Ochratoxin A + *Cupriavidus basilensis* ŐR16 in modified Luria- Bertani medium, LB bact- *Cupriavidus basilensis* ŐR16 in modified Luria- Bertani medium (Kruskal-Wallis test was used). OTA concentrations in the plasma (ng/ml) analyzed by ELISA after 21 days of treatment (**B**). The OTA 0.5 group shows elevated OTA blood concentrations. Abbreviations: MMS – Group treated with methyl methanesulfonate, OTA 0.5 – Group treated with 0.5 mg/kg body weight Ochratoxin A, OTA 0.5 deg – Group treated with 0.5 mg/kg body weight Ochratoxin A + *Cupriavidus basilensis* ŐR16 in modified Luria- Bertani medium, LB bact – *Cupriavidus basilensis* ŐR16 in modified Luria- Bertani medium. (One way ANOVA followed by the Tukey's *post hoc* test were used) Data are presented as mean ± SD (n = 7–10, **p<0.01)

Low levels of OTA were detected in the blood of vehicle or MMS-treated control animals (5.30± 3.9 and 3.20±4.9 ng/ml respectively). Acute mycotoxin treatment significantly elevated OTA concentration in the blood OTA 1 (269.73 ± 60.6 ng/ml OTA in blood plasma, p = 0.0055), and OTA 10 (1969.28 ±654.6 ng/ml OTA in blood plasma, p = 0.0023) groups. However, significantly lower levels of OTA were detected in plasma samples of mice treated with 10 mg/kg bw biodegraded OTA products in acute tests (101.18±11.4 ng/ml OTA, p = 0.0236) (Fig. 2A). Chronic (21 days) OTA treatment (0.5 mg/kg bw) resulted in 231.35± 50.23 ng/ml OTA level in the blood (p =  0.0001), however that elevation of OTA levels were not seen in animals treated with the same dose of biodegraded OTA (Fig. 2B). The batches of high quality animal feed were also tested by SFH laboratory (Soft Flow Hungary Ltd., Hungary), and traces of OTA were detected (3.05±0.12 µg/kg feed).

### 3.4 Effect of OTA on the spleen and kidney weight

Spleen and kidney are sensitive indicators of OTA toxicity. The acute OTA administration did not influence significantly the kidney wet weight, but the spleen wet weights normalized to body weight decreased significantly in both MMS and OTA treated groups (p = 0.0096, p = 0.0109 and p = 0,0393) (Fig. S1B in Supplementary Materials). On the other hand chronic OTA administration decreased significantly the kidney wet weight normalized to body weight only in the OTA treated group (p = 0.0059) (Fig. S1C in Supplementary Materials). The spleen wet weight normalized to body weight did not show statistically significant differences between the MMS and OTA treated groups (Fig. S1D in Supplementary Materials). The bacterial residuals did not showed toxic effects on the spleen and the kidney in both experiments as showed by their wet weight normalized to body weight.

### 3.5 Histopathological analysis of renal cortex tissue

**Figure 3:**
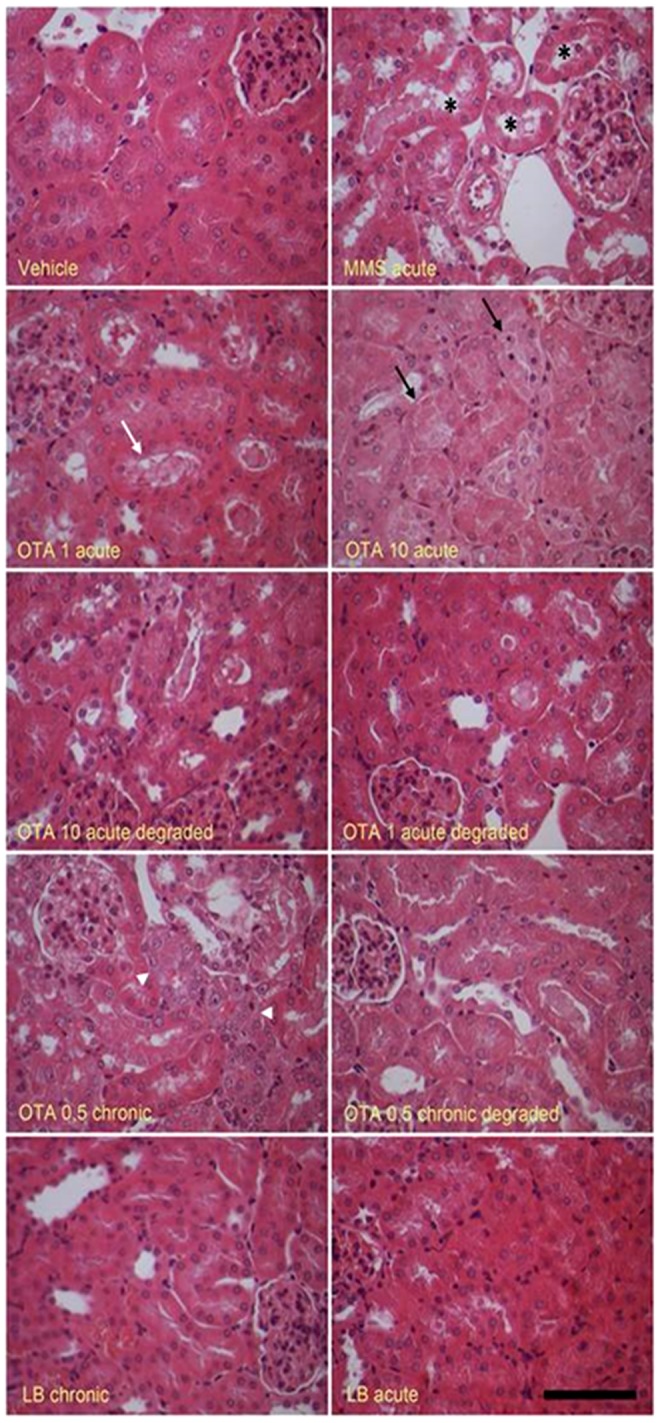
Histology of the kidneys following OTA and degraded OTA administrations. Photomicrographs showing hematoxylin-eosine stained kidney sections. Abbreviations: MMS- –methyl methanesulfonate treated animals, OTA 1 and OTA 10 – Groups treated with 1 and 10 mg/kg body weight Ochratoxin A, OTA 1 deg and OTA 10 deg – Groups treated with 1 and 10 mg/kg body weight Ochratoxin A + *Cupriavidus basilensis* ŐR16 in modified Luria-Bertani medium, OTA 0.5 – Group treated with 0.5 mg/kg body weight Ochratoxin A, OTA 0.5 deg – Group treated with 0.5 mg/kg body weight Ochratoxin A + *Cupriavidus basilensis* ŐR16 in modified Luria-Bertani medium, LB bact – *Cupriavidus basilensis* ŐR16 in modified Luria- Bertani medium. Symbols: asterisk- dilated tubules with detached necrotic epithelial cells, white arrow- detached necrotic epithelial cells, black arrow- necrotic tubular cells, white arrowhead- tubular cell regeneration. Scale bar: 100 µm.

Animals treated with OTA (1 mg/kg bw or 10 mg/kg bw) for 72 h showed clear degenerative lesions mainly located in the inner part of the cortex. Sporadic cell necrosis of the tubular epithelium with cell detachment to the tubular lumen was detectable. Multifocal tubular necrosis also occurred. Dispersed apoptotic bodies, cell size reduction and condensed chromatin in nucleus were observed at high OTA dosed groups and in the chronically treated animals. Beyond degenerative changes tubular cell regeneration has been detected in the chronic OTA treated group. Mice treated with biodegraded OTA and their residuals did not exhibit remarkable histopathological changes. The *Cupriavidus basilensis* Őr16 alone showed similar histology to vehicle group (Fig. 3).

### 3.6 Effect of acute OTA and OTA biodegradation residuals on the expression of various marker genes in the kidney

**Figure 4:**
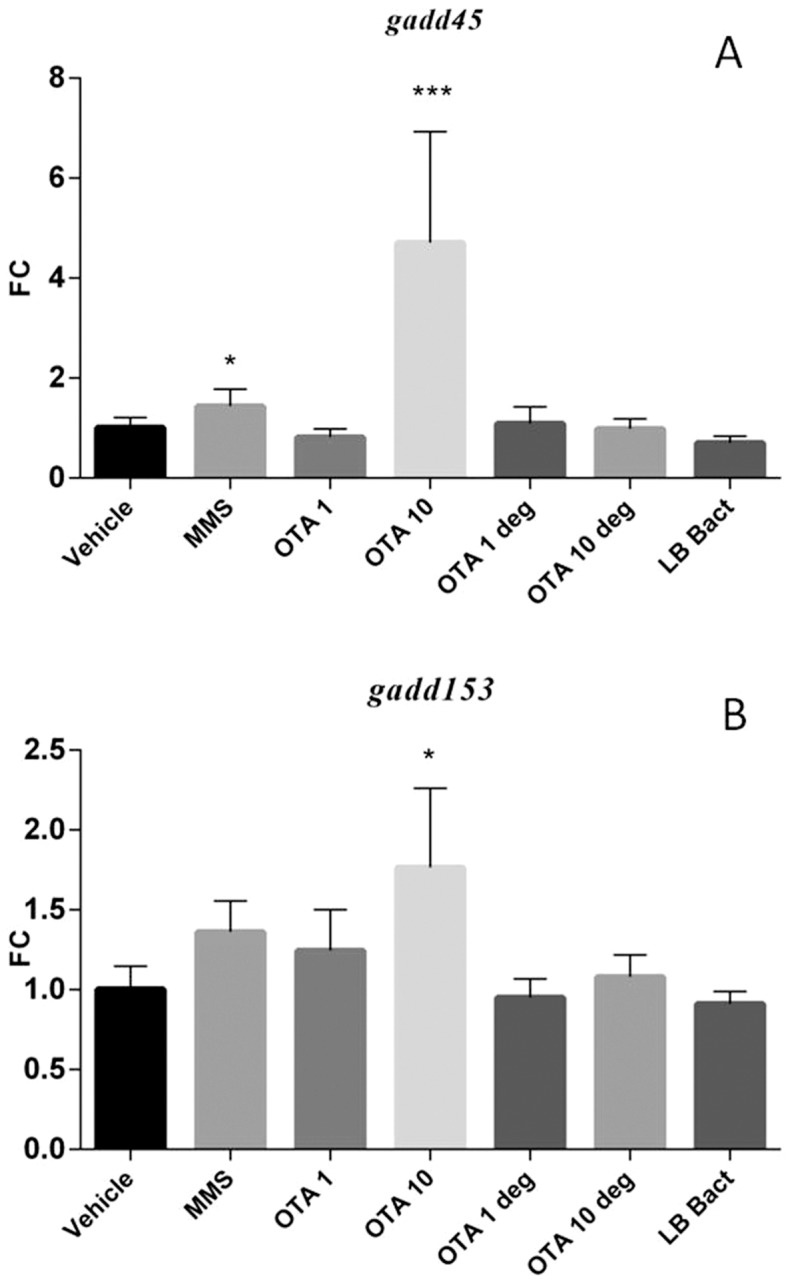
Effect of acute (72 hours) OTA treatment on *gadd45* mRNA expression (**A**). MMS and OTA 10 elevated the *gadd45* mRNA level. The metabolised OTA by *Cupriavidus basilensis* ŐR16 (OTA 1 deg and OTA 10 deg groups) not influenced the mRNA level (One way ANOVA followed by the Tukey's *post hoc* test were used). Effect of acute (72 hours) OTA treatment on *gadd153* mRNA expression (**B**). OTA 10 elevated the *gadd153* mRNA level. The metabolised OTA by *Cupriavidus basilensis* ŐR16 (OTA 1 deg and OTA 10 deg groups) not influenced the mRNA level. (Kruskal-Wallis test was used) Abbreviations: MMS – Group treated with methyl methanesulfonate, OTA 1 and OTA 10 – Groups treated with 1 and 10 mg/kg body weight Ochratoxin A, OTA 1 deg and OTA 10 deg – Groups treated with 1 and 10 mg/kg body weight Ochratoxin A + *Cupriavidus basilensis* ŐR16 in modified Luria-Bertani medium, LB bact- *Cupriavidus basilensis* ŐR16 in modified Luria-Bertani medium. Data are presented as mean ± SD (n = 7–10, * p<0.05, ***p<0.001)

**Figure 5:**
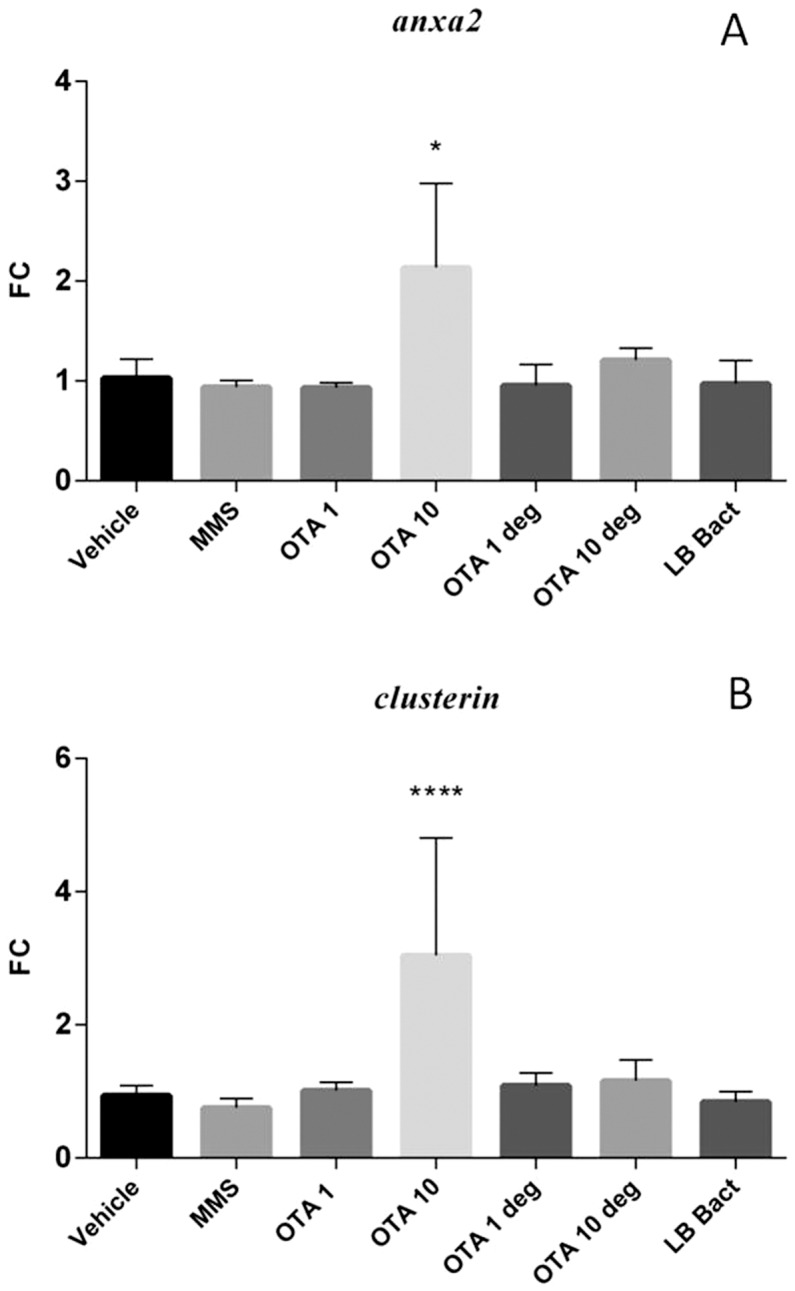
Effect of acute (72 hours) OTA treatment on *annexin2* mRNA expression (**A**). OTA 10 elevated the *annexin2* mRNA level. The metabolised OTA by *Cupriavidus basilensis* ŐR16 (OTA 1 deg and OTA 10 deg groups) did not influence the mRNA level (Kruskal-Wallis test was used). Effect of acute (72 hours) OTA treatment on *clusterin* mRNA expression (**B**). OTA 10 elevated the *clusterin* mRNA level. The metabolised OTA by *Cupriavidus basilensis* ŐR16 (OTA 1 deg and OTA 10 deg groups) did not influence the mRNA level. (One way ANOVA followed by the Tukey's *post hoc* test were used) Abbreviations: MMS – Group treated with methyl methanesulfonate, OTA 1 and OTA 10 – Groups treated with 1 and 10 mg/kg body weight Ochratoxin A, OTA 1 deg and OTA 10 deg- Groups treated with 1 and 10 mg/kg body weight Ochratoxin A + *Cupriavidus basilensis* ŐR16 in modified Luria-Bertani medium, LB bact – *Cupriavidus basilensis* ŐR16 in modified Luria-Bertani medium. Data are presented as mean ± SD (n = 7–10, ** p<0.01, *** p<0.001).

**Figure 6:**
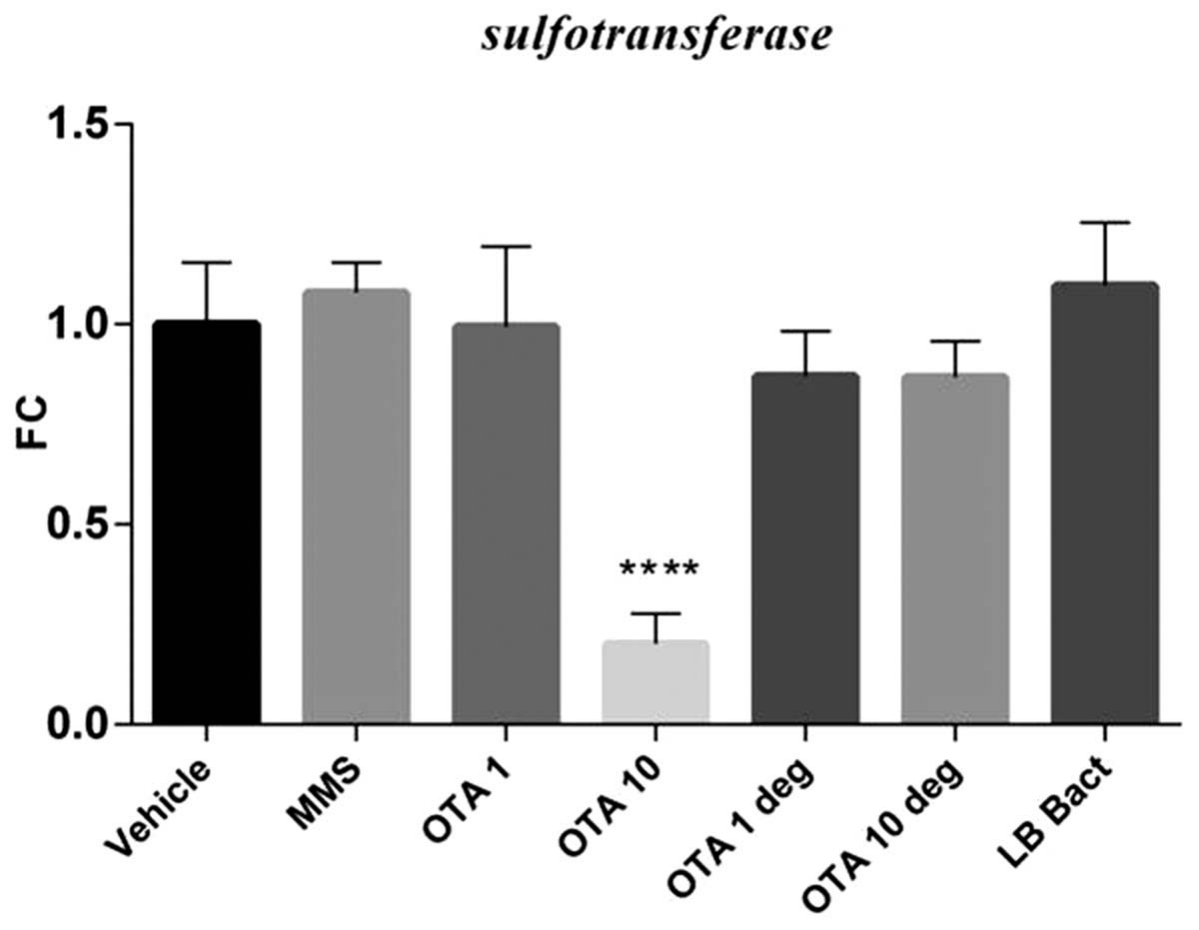
Effect of acute (72 hours) OTA treatment on *sult1c2* mRNA expression. OTA 10 elevated the *sult1c2* mRNA level alone. The metabolised OTA by *Cupriavidus basilensis* ŐR16 (OTA 1 deg and OTA 10 deg groups) not influenced the mRNA level. (One way ANOVA followed by the Tukey's *post hoc* test were used) Abbreviations: MMS- Group treated with methyl methanesulfonate, OTA 1 and OTA 10- Groups treated with 1 and 10 mg/kg body weight Ochratoxin A, OTA 1 deg and OTA 10 deg- Groups treated with 1 and 10 mg/kg body weight Ochratoxin A + *Cupriavidus basilensis* ŐR16 in modified Luria- Bertani medium, LB bact- *Cupriavidus basilensis* ŐR16 in modified Luria- Bertani medium. Data are presented as mean ± SD (n = 7–10, **** p<0.0001)

The high dose OTA and the MMS treatment significantly increased *gadd 45* (p =  0.00014) and *gadd 153* (p =  0.0112) mRNA levels (Fig. 4 A and B). The *Cupriavidus basilensis* ŐR16- metabolized OTA residuals did not influence mRNA levels of marker genes. LB with the bacterial strain did not change the expressions of genotoxic marker genes. The acute OTA administration with high dosage significantly elevated the *annexin2* mRNA expression (p =  0.0101). The end products of the OTA biodegradation did not change the *annexin2* mRNA expression in the kidney cortex samples (Fig. 5A). Elevated expression level of *clusterin* was observed in the high dose OTA treated group (p = 0.000141), however metabolites of the biodegraded OTA did not influence the *clusterin* mRNA levels (Fig. 5B). Animals treated with high OTA dosage showed significant decrease in the *sult1c2* mRNA levels (p =  0.000129). The biodegraded OTA did not influence the *sult1c2* mRNA expression in the renal cortex (Fig. 6).

### 3.7 Effect of chronic OTA and OTA biodegradation residuals on the expression of marker genes in the kidney

**Figure 7:**
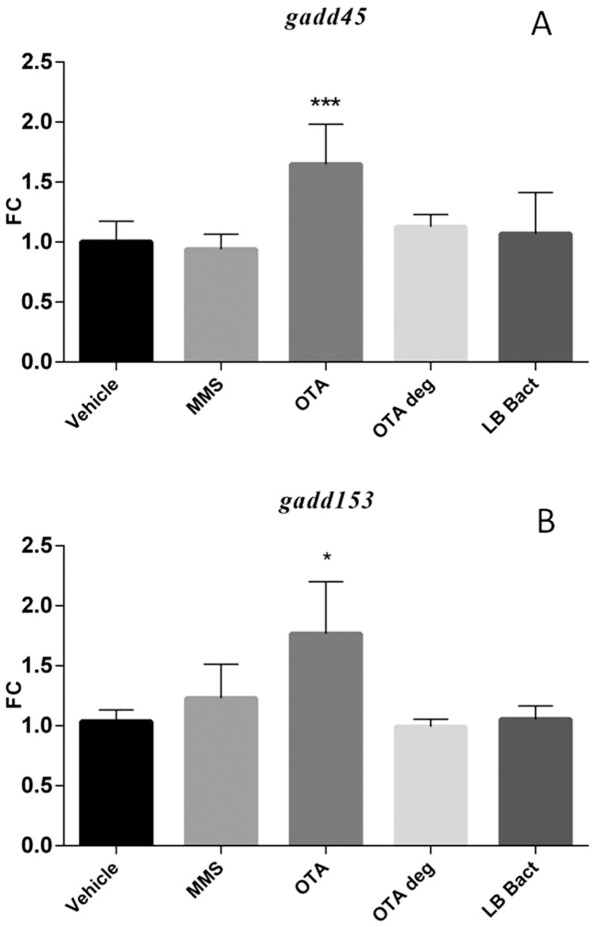
Effect of chronic (21 days) OTA treatment on *gadd45* mRNA expression (**A**). The OTA 0.5 mg/kg bw chronic administration elevated the *gadd45* mRNA level. The metabolised OTA by *Cupriavidus basilensis* ŐR16 (OTA 0.5 deg group) not influenced the mRNA levels (One way ANOVA followed by the Tukey's *post hoc* test were used). Effect of chronic (21 days) OTA treatment on *gadd153* mRNA expression (**B**). The OTA 0.5 mg/kg bw chronic administration elevated the *gadd153* mRNA level. The metabolised OTA by *Cupriavidus basilensis* ŐR16 (OTA 0.5 deg group) did not influence the mRNA level (Kruskal-Wallis test was used). Abbreviations: MMS – Group treated with methyl methanesulfonate, OTA 0.5 – Group treated with 0.5 mg/kg body weight Ochratoxin A, OTA 0.5 deg – Group treated with 0.5 mg/kg body weight Ochratoxin A + *Cupriavidus basilensis* ŐR16 in modified Luria-Bertani medium, LB bact – *Cupriavidus basilensis* ŐR16 in modified Luria-Bertani medium. Data are presented as mean ± SD (n = 7–9, *p<0.05***p<0.001)

**Figure 8:**
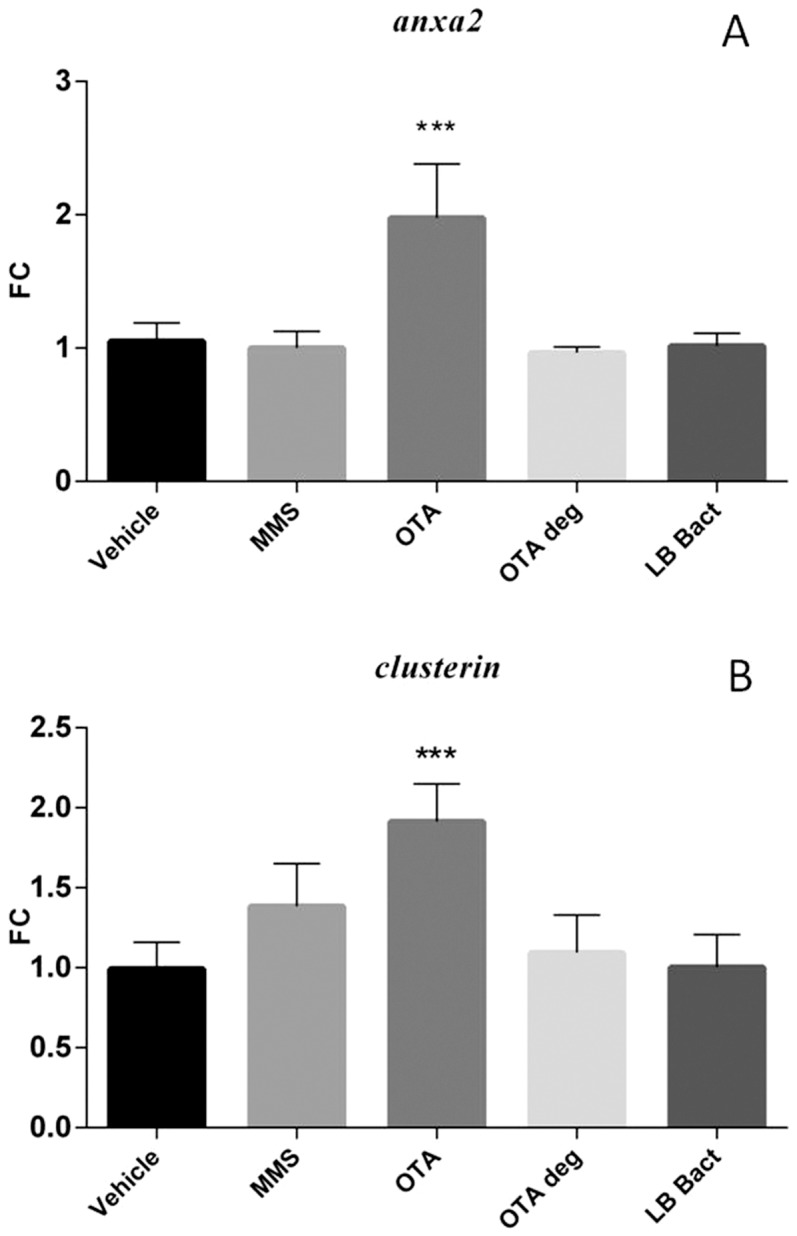
Effect of chronic (21 days) OTA treatment on *annexin2* mRNA expression (**A**). The OTA 0.5 mg/kg bw chronic administration elevated the *annexin2* mRNA level. The metabolised OTA by *Cupriavidus basilensis* ŐR16 (OTA 0.5 deg group) did not influence the mRNA level. (One way ANOVA followed by the Tukey's *post hoc* test were used) Effect of chronic (21 days) OTA treatment on *clusterin* mRNA expression (**B**). The MMS and OTA 0.5 mg/kg bw chronic administration elevated the *clusterin* mRNA level. The metabolised OTA by *Cupriavidus basilensis* ŐR16 (OTA 0.5 deg group) did not influence the mRNA level. (One way ANOVA followed by the Tukey's *post hoc* test were used) Abbreviations: MMS – Group treated with methyl methanesulfonate, OTA 0.5 – Group treated with 0.5 mg/kg body weight Ochratoxin A, OTA 0.5 deg – Group treated with 0.5 mg/kg body weight Ochratoxin A + *Cupriavidus basilensis* ŐR16 in modified Luria-Bertani medium, LB bact – *Cupriavidus basilensis* ŐR16 in modified Luria- Bertani medium. Data are presented as mean ± SD (n = 7–9, *** p<0.001)

**Figure 9:**
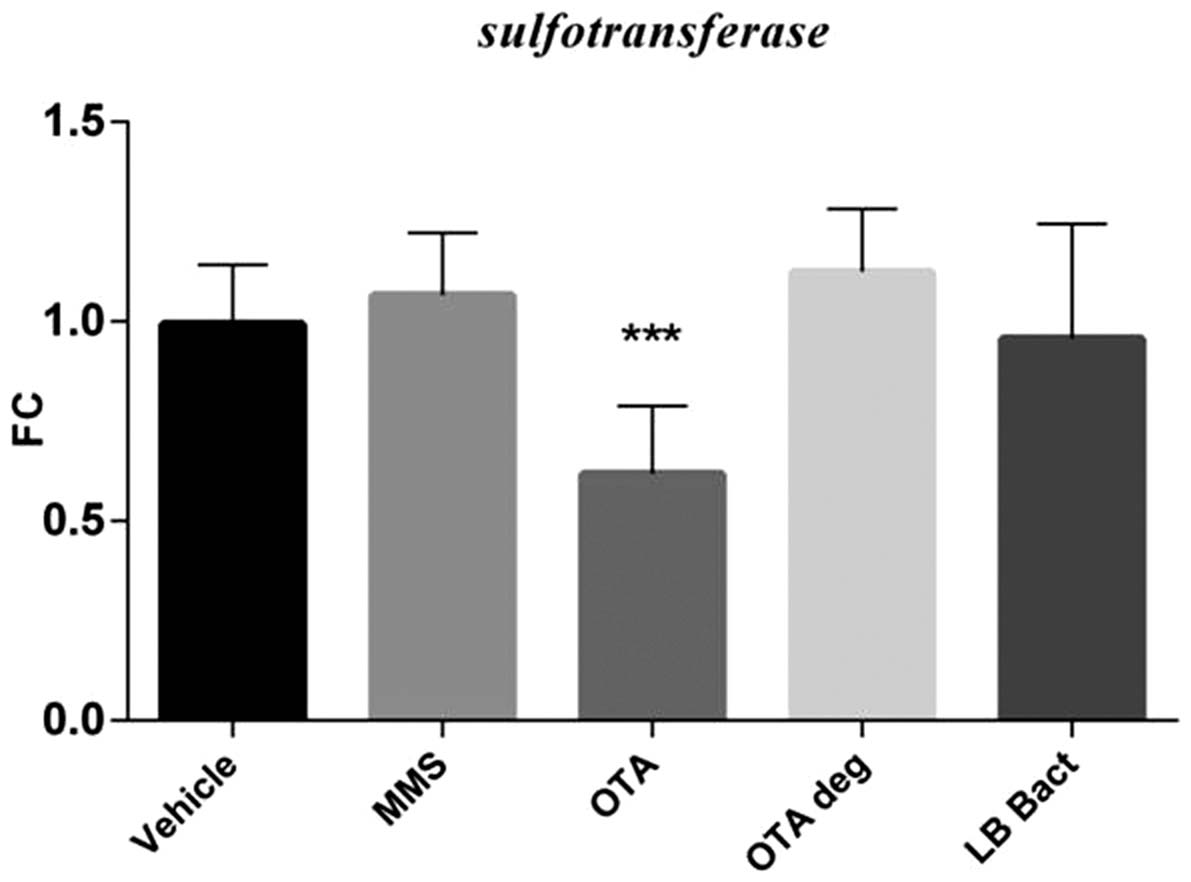
Effect of chronic (21 days) OTA treatment on *sult1c2* mRNA expression. The OTA 0.5 mg/kg bw chronic administration elevated the *sult1c2* mRNA level. The metabolised OTA by *Cupriavidus basilensis* ŐR16 (OTA 0.5 deg group) did not influence the mRNA level. (One way ANOVA followed by the Tukey's *post hoc* test were used) Abbreviations: MMS – Group treated with methyl methanesulfonate, OTA 0.5 – Group treated with 0.5 mg/kg body weight Ochratoxin A, OTA 0.5 deg- Group treated with 0.5 mg/kg body weight Ochratoxin A + *Cupriavidus basilensis* ŐR16 in modified Luria-Bertani medium, LB bact- *Cupriavidus basilensis* ŐR16 in modified Luria-Bertani medium. Data are presented as mean ± SD (n = 6–9, **p<0.01)

The chronic low dose OTA exposure significantly induced *gadd45* (p = 0.0001) and *gadd153* (p =  0.0141) mRNA levels in the kidney. MMS and OTA degradation products did not affect the mRNA levels of the genotoxic markers (Fig. 7A and 7B). The *annexin2* expression was increased in OTA treated animals (p =  0.000177) (Fig. 8A). On the other hand *clusterin* mRNA expression was up-regulated in the MMS (non significant) and OTA 0.5 (p = 0.0002) groups. The degraded OTA remnants did not affect *clusterin* expression (Fig. 8B). In contrast, the *sult1c2* mRNA expression was decreased significantly (p = 0.0005) in the renal cortex during the OTA administration in the chronic experiment; while the biodegraded OTA did not show effect on the *annexin2* and *sult1c2* mRNA expressions in the kidney (Fig. 8A and Fig. 9).

## Discussion

Several studies on OTA degrading, adsorbing and detoxifying agents have been published recently. Certain *Lactobacillus* species have moderate OTA degradation capacity however, the toxicity of the metabolites remains unclear [25]. Moreover, *Bacillus licheniformis* degraded 92.5% of OTA at 37°C and OTα, as degradation product, was detected [26]; while *Brevibacterium spp*. strains showed 100% degradation of OTA [27]. Furthermore an OTA degrading enzyme was identified in *Aspergillus niger*, which is capable to metabolize the OTA to phenylalanine and OTα [28]. Some *Trichosporon* species have the ability to cleave OTA selectively into phenylalanine and OTα but then the presumed nephrotoxic effect of other alternative metabolites were not investigated at gene expression level [29]. *Trichosporon mycotoxinivorans* was classified as a novel species due to ability to detoxify OTA [30]. This yeast, when introduced to the diet of broiler chickens completely abolished OTA effects on the immune system [31]. However, a recent study was raising doubts over the safety of *T. mycotoxinivorans* as it was associated with cystic fibrosis and the death of a patient with histological documented *Trichosporon* pneumonia [32].

**Figure 10:**
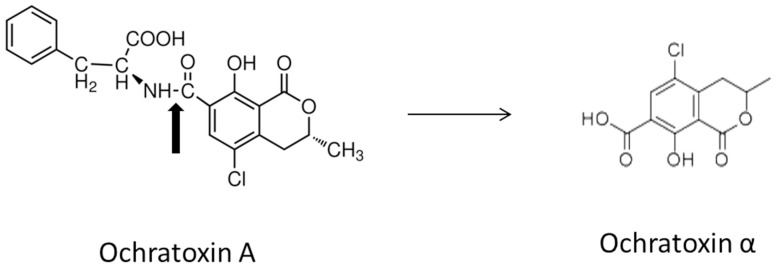
Proposed cleavage of Ochratoxin A by *Cupriavidus basilensis* ŐR16. The amide bond hydrolysis (up arrow) resulting Ochratoxin α as a major degradation product.

In our experiment the OTA-degrading potential of *C. basilensis* ŐR16 and acute/chronic toxicity of the degraded products were analysed. The OTA was degraded efficiently by the 5^th^ day of biodegradation. Moreover, metabolised form of OTA was also measured. Two pathways may be involved in OTA microbiological degradation. First, OTA can be biodegraded through the hydrolysis of the amide bond that links the L-*β*-phenylalanine molecule to the OTα moiety. Since OTα and L-*β*-phenylalanine are virtually non-toxic, this mechanism can be considered to be a detoxification pathway. Second, a more hypothetical process involves OTA being degraded via the hydrolysis of the lactone ring [33]. In this case, the final degradation product is an opened lactones form of OTA, which has similar toxicity than OTA when administered to rats [34], [35]. In the present study OTα was detected by HPLC, and also the time-curve detected increasing OT-α concentration parallel with the OTA-decrease. Based on this phenomenon cleavage of the amide bond that links the L-*β*-phenylalanine molecule to the OTα moiety is hypothesized (Fig. 10). This observation was confirmed by *in vivo* toxicological tests.

The major metabolite of the bacterial OTA degradation is the OTα, which is a non-toxic metabolite of the OTA but then formations of other toxic residuals are possible [36], [37]. Chemical analysis and immunochemical methods are less sensitive to detect all potential harmful degradation products, therefore it is important to monitor for toxicity by *in vivo* methods.

After oral administration of various doses of OTA in acute and chronic experiments, significant elevation of the OTA concentrations were found in plasma. Even in control animals OTA levels above the detection limit were found, which could be attributed to natural contamination, as it was reported previously [38]–[40]. OTA concentration in the plasma showed positive correlation with the administered mycotoxin doses. On the other hand elevated OTA concentration was detected in the blood samples after acute exposure with high doses of biodegraded OTA metabolites; however, this OTA concentration was only 5.1 % compared to the OTA level detected in the OTA 10 group (Fig. 2A). Presumably the residual non degraded OTA was accumulated in the blood. This result was confirmed by ELISA tests which detected 94% biodegradation efficiency of the *Cupriavidus basilensis* ŐR16 (Fig. 1).

The long-term OTA exposure during the chronic mycotoxin treatment significantly decreased the kidney weight but then the acute treatment with the detoxified metabolites of OTA did not affect it, which alteration is the most toxins sensitive. These observations were established by previous reports in which chronic OTA exposure induced nephropathy in animals and humans, as well [8], [12], [13]. The OTA degradation by *Cupriavidus basilensis* ŐR16 completely abolished these effects. This phenomenon was confirmed by histopathology analysis. The high dose of acute and the lower dose of chronic OTA treatment induced toxicities related malformations in renal cortex tissue (Fig. 3). These results were also detected in previous studies of Lühe and co-workers in acute experiments and also in chronic experiments on rodent models by Rached and colleagues [19], [41]. In the present work, neither the *Cupriavidus basilensis* ŐR16 with modified LB, nor the degraded OTA residuals formed pathological disorders.

The spleen, the other mycotoxin sensitive organ, was examined with different OTA dosages in acute and chronic experiments. The spleen weight was significantly decreased after acute OTA and MMS exposure, but the chronic OTA and MMS treatments did not decrease the spleen weight significantly. The rapid OTA accumulation in the blood after high dosage treatment could explain the suppression of immune system and the lymphoid organ weight loss. Similar observations were published in case of broiler chickens after chronic OTA administration [42], [43]. Moreover, by analysing blood samples and lymphoid organs of humans and other mammalians, altered immune functions were also detected [44]. Histopathological examinations were demonstrated that the immunotoxic effect and histological malformation regarding the degraded OTA residuals and the *Cupriavidus basilensis* ŐR16 strain was not detected at histopathological level. Our bacterial OTA degradation strategy is an effective detoxification process by toxicological and physiological point of view.

Previous molecular genetic analysis demonstrated that the OTA influences the expression of genotoxic, apoptotic, detoxification and inflammation related genes in a dose dependent manner. This study revealed 254 genes which expression changed by at least two-fold after acute (3 day) OTA exposure (1 and 10 mg/kg bw). Eighty-nine genes were up regulated and 165 were down regulated. In the present work dose and time dependent Discriminator Genes were selected [19]. These genes are useful markers in the renal cortex for monitoring possible toxic effects of the end products after biodegradation. The selected, robust up- or down-regulated marker genes along with the adjusted experimental circumstances encompass the complete toxic action of OTA and the applied indicators are sensitive for many of the possible toxicities of the bio-converted OTA residuals. The expressions of OTA induced marker genes were detected by quantitative-real-time-PCR measurement; since one of the major effects of OTA is the DNA damaging action [45]. The *gadd 45* and *gadd 153* genes serves as markers to demonstrate the genotoxic effect and DNA damage induction of OTA, besides these genes are monitoring the biodegradation of OTA. By the degradation of OTA, the toxicity is eliminating which results unaltered *gadd 45* and *gadd 153* mRNA levels. MMS treated animals served as positive control, because MMS is a well-studied genotoxic chemical that induces alterations in *gadd 45* and *gadd 153* mRNA expressions [46]. While OTA up-regulated the expression of *gadd 45* and *gadd 153* in a dose dependent manner, the metabolised OTA by *Cupriavidus basilensis* ŐR16 completely eliminated this up-regulation in both experiments. Furthermore, the OTA is involved in renal tumour formation. The *clusterin* and *annexin2* play important role in the apoptotic processes and in the renal tumour formation [47]. Previous studies have demonstrated *annexin 2* (*anxa2*) up-regulation by acute OTA treatment in the renal cortex of rats, which resulted similar mRNA expression level that was described in our acute experiment in mice [19]. *Annexin2* is a cofactor for DNA polymerase alpha subunit that plays an important role in the DNA repair and in the development of different cancers [48]. Furthermore *annexin2* is a substrate for an oncogene associated kinase [49]. Elevated level of *annexin2* was described in the kidney carcinoma formation in rat [50]. The OTA biodegradation by *Cupriavidus basilensis* ŐR16 strain abolished the up-regulation of both marker genes in both acute and chronic treatments, indicating that the biodegradation was a detoxification as well.

*Sulfotransferase 1c2* serves as a marker for the cellular and detoxification processes, which has been identified as an OTA-induced gene in acute (72 hours) mycotoxin treatment [19]. *Sulfotransferase* takes part in xenobiotic detoxification, carcinogen activation, prodrug processing, and cellular signalling pathways [51]. Our work demonstrated that *sulfotransferase 1c2* expression decreased after acute and chronic OTA exposure. OTA biodegradation by *Cupriavidus basilensis* ŐR16 strain abolished the *sulfotransferase* mRNA down-regulation in both experiments.

Based on our present results, the major metabolite of OTA biodegradation by *Cupriavidus basilensis* ŐR16 is Ochratoxin α. This metabolite is not toxic *in vitro* and here we found that biodegradation product does not display nephrotoxic effects *in vivo*. In summary, by application of *Cupriavidus basilensis* ŐR16, OTA is degraded efficiently without bioactive intermediates and by-products; therefore *Cupriavidus basilensis* ŐR16 is worth further study for possible use for the decontamination of raw materials, to reduce OTA concentration.

## Supporting Information

Figure S1 The acute OTA administration did not influence significantly the kidney normalized weight (**A**). (Kruskal-Wallis test was used). The acute OTA administration significantly decreased the spleen normalized weights of animals in the MMS, OTA 1 and OTA 10 groups (**B**). (Kruskal-Wallis test was used). Abbreviations: MMS – Group treated with methyl methanesulfonate, OTA 1 and OTA 10 – Groups treated with 1 and 10 mg/body weight kg Ochratoxin A, OTA 1 deg and OTA 10 deg- Groups treated with 1 and 10 mg/body weight kg Ochratoxin A + *Cupriavidus basilensis* ŐR16 in modified Luria-Bertani medium, LB bact - *Cupriavidus basilensis* ŐR16 in modified Luria-Bertani medium. Data are presented as mean ± SD (n = 7-10, *p<0.05,**p<0.01,). The chronic OTA administration significantly decreased the kidney normalized weight of animals in the OTA 0.5 groups (**C**). (One way ANOVA followed by the Tukey's *post hoc* test were used). The chronic OTA administration decreased the spleen normalized weight of animals in the MMS and OTA 0.5 groups (**D**). The alterations were not significant. (Kruskal-Wallis test was used). Abbreviations: MMS – Group treated with methyl methanesulfonate, OTA 0.5 – Group treated with 0.5 mg/body weight kg Ochratoxin A, OTA 0.5 deg – Group treated with 0.5 mg/body weight kg Ochratoxin A + *Cupriavidus basilensis* ŐR16 in modified Luria-Bertani medium, LB bact – *Cupriavidus basilensis* ŐR16 in modified Luria- Bertani medium. Data are presented as mean ± SD (n = 7-10, **p<0.01).
(TIF)

Table S1 **Detailed information about analytical methods for ochratoxin-A, and its derivative ochratoxin-α.**
(DOCX)

Table S2 **The nucleotide sequence of the oligonucleotid primers.**
(DOCX)
