# Supplementary figures and images for: A New Ochratoxin A Biodegradation Strategy Using Cupriavidus basilensis Őr16 Strain
Source: PLoS One. 2014 Oct 10;9(10):e109817. doi: 10.1371/journal.pone.0109817 (PMC4193827; doi:10.1371/journal.pone.0109817)

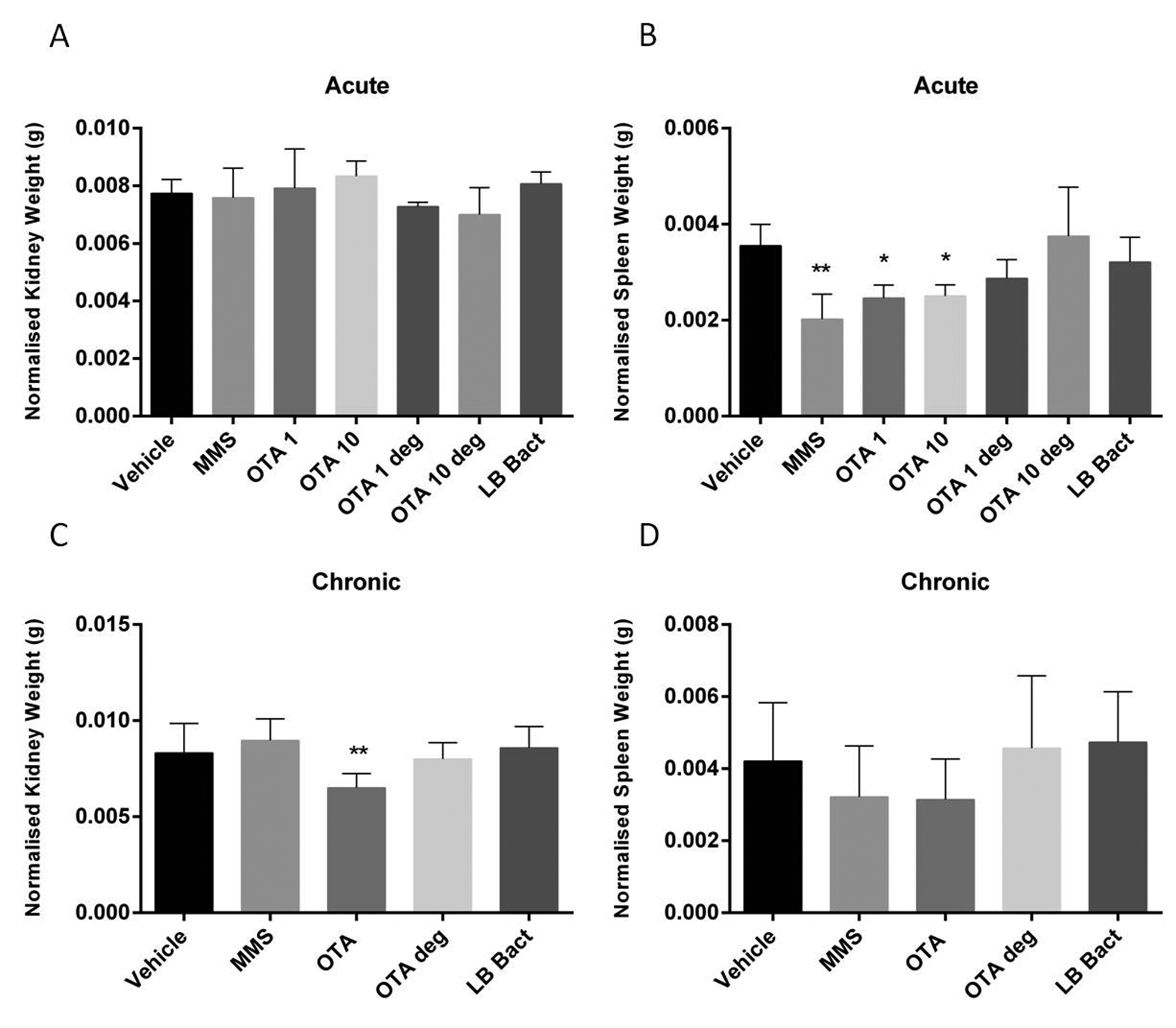

Supplement: Figure S1 — The acute OTA administration did not influence significantly the kidney normalized weight (A). (Kruskal-Wallis test was used). The acute OTA administration significantly decreased the spleen normalized weights of animals in the MMS, OTA 1 and OTA 10 groups (B). (Kruskal-Wallis test was used). Abbreviations: MMS – Group treated with methyl methanesulfonate, OTA 1 and OTA 10 – Groups treated with 1 and 10 mg/body weight kg Ochratoxin A, OTA 1 deg and OTA 10 deg- Groups treated with 1 and 10 mg/body weight kg Ochratoxin A + Cupriavidus basilensis ŐR16 in modified Luria-Bertani medium, LB bact - Cupriavidus basilensis ŐR16 in modified Luria-Bertani medium. Data are presented as mean ± SD (n = 7-10, *p<0.05,**p<0.01,). The chronic OTA administration significantly decreased the kidney normalized weight of animals in the OTA 0.5 groups (C). (One way ANOVA followed by the Tukey's post hoc test were used). The chronic OTA administration decreased the spleen normalized weight of animals in the MMS and OTA 0.5 groups (D). The alterations were not significant. (Kruskal-Wallis test was used). Abbreviations: MMS – Group treated with methyl methanesulfonate, OTA 0.5 – Group treated with 0.5 mg/body weight kg Ochratoxin A, OTA 0.5 deg – Group treated with 0.5 mg/body weight kg Ochratoxin A + Cupriavidus basilensis ŐR16 in modified Luria-Bertani medium, LB bact – Cupriavidus basilensis ŐR16 in modified Luria- Bertani medium. Data are presented as mean ± SD (n = 7-10, **p<0.01). (TIF) [file pone.0109817.s001.tif]
