# Supplementary material for: A New Ochratoxin A Biodegradation Strategy Using Cupriavidus basilensis Őr16 Strain
Source: PLoS One. 2014 Oct 10;9(10):e109817. doi: 10.1371/journal.pone.0109817 (PMC4193827; doi:10.1371/journal.pone.0109817)
Supplement: Table S1 — Detailed information about analytical methods for ochratoxin-A, and its derivative ochratoxin-α. (DOCX) [file pone.0109817.s002.docx]

Supplementary Materials

Table S1: Detailed information about analytical methods for ochratoxin-A, and its derivative ochratoxin-α

|  | **Ochratoxin-A** | **Ochratoxin-α** |
| --- | --- | --- |
| Method | MSZ EN ISO 15141-1:2000 | modified method for OTA |
| Instrument | Agilent 1100 HPLC-FLD | Agilent 1100 HPLC-FLD |
| Column | Vydec Denali | Agilent Zorbax Eclipse XDB-C18 |
| Eluent | water / acetonitril / acetic acid (49.5:49.5:1) | water / acetonitril / acetic acid (69.5:29.5:1) |
| Detection wavelength | excitation: 330 nm  emission: 460 nm | excitation: 333 nm  emission: 436 nm |
| Limit of quantification | 2 μg/l | 200 μg/l |
| Limit of detection | 0.3 μg/l | 30 μg/l |
