# Supplementary material for: A New Ochratoxin A Biodegradation Strategy Using Cupriavidus basilensis Őr16 Strain
Source: PLoS One. 2014 Oct 10;9(10):e109817. doi: 10.1371/journal.pone.0109817 (PMC4193827; doi:10.1371/journal.pone.0109817)
Supplement: Table S2 — The nucleotide sequence of the oligonucleotid primers. (DOCX) [file pone.0109817.s003.docx]

Supplementary Materials

Table S2. The nucleotide sequence of the oligonucleotid primers

| gene names | oligo sequence |
| --- | --- |
| ppia f | CGCGTCTCCTTCGAGCTGTTTG |
| ppia r | TGTAAAGTCACCACCCTGGCACA |
| anxa2 f | GACATTGCCTTCGCCTATCA |
| anxa2 r | GCCAGATAAGGCTGACTTCAG |
| clu f | CACTATGGGCCTCCGAGCTT |
| clu r | ACATCCATGGCCTGTTGAG |
| gadd153 f | CTGCCTTTCACCTTGGAGAC |
| gadd153 r | CCCCAATTTCATCTGAGGACA |
| gadd45a f | GCCAAGCTGCTCAACGTAGAC |
| gadd45a r | AGGGTGAAATGGATCTGCAGA |
| sult1c2 f | GCCAGAAATACGTCCCTCCT |
| sult1c2 r | GTCCAGTCCCTTCAAGCTCT |
